# Mountain Hiking vs. Forest Therapy: A Study Protocol of Novel Types of Nature-Based Intervention

**DOI:** 10.3390/ijerph19073888

**Published:** 2022-03-24

**Authors:** Christina Pichler, Johanna Freidl, Michael Bischof, Martin Kiem, Renate Weißböck-Erdheim, Daniela Huber, Gabriella Squarra, Paul Clemens Murschetz, Arnulf Hartl

**Affiliations:** 1 Institute of Ecomedicine, Paracelsus Medical University, 5020 Salzburg, Austria; christina.pichler@pmu.ac.at (C.P.); johanna.freidl@pmu.ac.at (J.F.); michael.bischof@pmu.ac.at (M.B.); renate.erdheim@pmu.ac.at (R.W.-E.); daniela.huber@pmu.ac.at (D.H.); paul.murschetz@pmu.ac.at (P.C.M.); 2 Certified Nature and Forest Therapy Guide, 39010 Tisens, Italy; info@martin-kiem.com; 3 Certified Forest-Health-Trainer, 83435 Bad Reichenhall, Germany; squarra.gabriella@googlemail.com

**Keywords:** nature and health, green exercise, forest therapy, sedentary lifestyle, climate therapy, health-related quality of life (HRQOL), intercultural quality of life assessment

## Abstract

Introduction: The global rise of urbanization has much triggered scientific interest in how nature impacts on human health. Natural environments, such as alpine landscapes, forests, or urban green spaces, are potential high-impact health resources. While there is a growing body of evidence to reveal a positive influence of these natural environments on human health and well-being, further investigations guided by rigorous evidence-based medical research are very much needed. Objective: The present study protocol aims at testing research methodologies in the context of a prospective clinical trial on nature-based interventions. This shall improve the standards of medical research in human–nature interactions. Methods: The ANKER Study investigates the influence of two novel types of nature-based therapy—mountain hiking and forest therapy—on physiological, psychological, and immunological parameters of couples with a sedentary lifestyle. Two intervention groups were formed and spent a seven-day holiday in Algund, Italy. The “forest therapy group” participated in daily guided low-power nature connection activities. The “hiking group”, by contrast, joined in a daily moderate hiking program. Health-related quality of life and relationship quality are defined as primary outcomes. Secondary outcomes include nature connection, balance, cardio-respiratory fitness, fractional exhaled nitric oxide, body composition and skin hydration. Furthermore, a new approach to measure health-related quality of life is validated. The so-called “intercultural quality of life” comic assesses the health-related quality of life with a digitally animated comic-based tool.

## 1. Introduction

Over a whole lifetime, human health is dynamically affected by a plethora of external and internal factors. Following the Exposome concept, both external push factors and internal factors that are influenced directly or more indirectly by humans themselves play a seminal role in health development over time [1,2,3,4]. As has been shown elsewhere, genetic predisposition may affect the personal risk of developing chronic diseases by 10% [5]. This is a rather small factor in the present context. External or environmental factors, such as diet, exercise, place of residence, access to green space or climatic conditions, have been changing rapidly over the last centuries, leading to increasing numbers of non-communicable diseases [6]. Scientific evidence is growing that natural environments have a great potential for disease prevention and health promotion [7]. However, current research often lacks methodological quality and rarely meets state-of-the art criteria to assess the impact of natural environments as an external factor on human health and well-being [8].

How exactly and to what extent forests promote human health is currently only researched to a limited extent, although there is increasing evidence for a positive effect of forest therapies. This applies to both the physical, mental and social health of humans [9]. Schuh and Immich [10] find that forest therapies are particularly suitable for promoting the general health and act as stress reducers. Several studies on stress reduction through forest therapies show an increase in parasympathetic activity [11,12,13]. These play an important role in physical recovery and relaxation [14] but may also have a positive effect on blood pressure [15] and heart rate [16]. Furthermore, forest therapies can be used to mitigate known risk factors for cardiovascular diseases [17,18]. Respiratory diseases, such as COPD [19], depression [20], exhaustion [21] and sleep disorders [22], can also be positively influenced by forest-based interventions. Moreover, as evidenced, forests have positive effects on the immune system [11]. In general, more frequent and longer stays in forests have a stronger and more lasting effect than isolated and shorter visits [23]. However, a curative effect of forest stays on existing diseases has not been proven yet [10].

Overall, it can be said that, while there are numerous studies on health effects of forests, there is still much need for improved research conceptually, as well as methodologically [9]. Unfortunately, we have identified various studies on forest-based interventions with medium to poor quality and high risk of bias. Structural and methodological weaknesses in study design and reporting quality come with insufficient description of the intervention groups, reporting of confounding variables and missing or inadequate control group organizations [24]. Furthermore, most studies identified are performed in Asian countries with mostly healthy and young participants, thus limiting the generalizability of the results. The intervention duration is mostly rather short (1–3 days) without reporting long-term effects [25,26]. In addition, it must be taken into account that possible health effects of forests may be triggered by individual perceptions of forest visitors and thus by the setting of the forest itself [27]. Therefore, a homogenous assessment tool to characterize the forest itself would be particularly important. By structuring the numerous findings on the impact of forests, it is possible to derive a basic evaluation scheme for individual forest areas, which can be used as a comparative instrument between different study areas and, therefore, also contribute to the necessary improvement of the approach of the Exposome [8]. The characteristics of the forest or the trees can be divided into different categories, each with measurable indicators. These are:

(1) *Size of the forest area*: Larger, more coherent forests increase well-being and can also be activity enhancing [28].
(2) *Age of trees*: Older forests with large and mature trees increase well-being and positively contribute to recreational preferences [29].
(3) *Stock of trees*: Mixed forests with deciduous and coniferous trees are perceived as more attractive than monocultures and thus increase well-being [30].
(4) *Height and structure of the trees*: Higher trees increase well-being. In addition, different tree heights (levels of the treetops) are perceived as more attractive [27].
(5) *Stand density of the trees*: Light forests with a rather low stand density of trees, and thus a higher incidence of light, increase well-being [10].
(6) *Characteristics of the treetops*: A crown covering of about 75%, combined with sufficient light incidence, increases well-being [27].
(7) *Characteristics of the forest as a whole*: Well-tended forests in the sense of managed forests (mood-lifting effect) [31] and a low proportion of dead wood, but at the same time, no excessive traces of lumbering [27], are preferred. In addition, the forests should be bright (orientation and safety), free of waste and noise [27].
(8) *Other vegetation*: A varied, green-to-colorful vegetation (in addition to the trees), which is neither too dense nor too open, is generally preferred [14].

The effects of the forest floor can be described with the help of two categories: (1) “characteristics of the forest paths”; and (2) “characteristics of the forest floor”. Thus, the following statements and indicators can be derived from the literature:

(1) *Characteristics of the forest paths*: Flat, easily walkable paths, as well as free waysides and thus easy orientation (wide view), increase well-being, as well as the recreational value [32].
(2) *Characteristics of the forest floor*: An area-wide vegetation, which is not overgrown and essentially walkable, increases well-being [32].

Apart from the forest characteristics listed above, other natural and artificial elements impact on the well-being of forest visitors. Natural factors include the presence of water (e.g., creeks, rivers, lakes, waterfalls), natural resting places (e.g., moss, snags, meadows, mounds, clearings) and existing views or scenery [33]. Artificial elements include infrastructure, such as recreation areas (e.g., benches), the signage of the paths or possibilities for protection from the elements of nature (e.g., shelters). For infrastructural elements, suitable materials (e.g., wood and stone instead of plastic and metal) should be chosen, and an overloading of nature with artificial elements should be avoided [28,34,35]. Further, the issue of barrier-free access can also be important in the context of forest therapies [36].

Considering the multitude of indicators for the assessment of the forest as defined above, it can be assumed that not all factors classified as positive in the literature are always fulfilled or present. On the one hand, it is difficult to find the perfect forest area in terms of the listed criteria. On the other hand, numerous external factors, such as ownership or accessibility, also play an important role in the possible use of a forest area. In this respect, the forest area used is almost always a compromise solution between potential optimum and reality. It is therefore even more important to integrate the question of the actual effect of the forest setting into future studies. Thus, the forest as a natural space is also a factor that can have an influence on human health. Generally, the Exposome provides an interesting approach, especially for the health effects of our natural environment, which, in our view, requires intensified research and further methodological approaches [8]. 

### Objectives and Trial Design

The present paper represents a protocol of a prospective clinical study assessing the effects of two different nature-based interventions on human health and well-being, following the SPIRIT guidelines [37]. The purpose is to improve the levels of methodological quality in nature-based therapy research meeting validity criteria of reproducibility by other research groups. It will thus contribute to improving the standards of medical research regarding nature-based interventions and contribute to a more solid body of evidence regarding the linkage between nature and human health and well-being.

The objective of the ANKER Study (“Algunder Nature and climate therapy: Green Exercise vs. Nature Connection”) is to analyze effects of two types of nature-based interventions—(1) Hiking and (2) Forest Therapy—in couples with a sedentary lifestyle on health-related quality of life, quality of relationship, and further psychological and physiological parameters are investigated.

## 2. Materials and Methods 

### 2.1. Participants, Interventions and Outcomes

#### 2.1.1. Study Design

The ANKER Study was designed as a two-armed randomized controlled trial. It aimed at investigating the effects of moderate mountain hiking and forest therapy on couples with a sedentary lifestyle. Participants were assigned to two intervention groups: (1) a “hiking group”; and (2) a “forest therapy group”. Both groups spent a seven-day holiday in Algund, Italy, and participated in daily hiking or forest therapy activities. The study was carried out in two independent sequences but with the same intervention schedule. One half of the study population finished the ANKER Study in October 2019. The second study sequence was scheduled for April 2020, but due to the global COVID-19 pandemic, this part was only carried out in June 2021, when hotels in Algund re-opened, and a safe implementation was guaranteed.

#### 2.1.2. Eligibility Criteria

The ANKER Study included couples with a sedentary lifestyle presenting the following demographics: age 50–60 years old, relationship duration >1 year, body mass index ≥25–≤30, sedentary lifestyle (International Physical Activity Questionnaire Short Form <3.00 METmin/week) and the ability to participate in moderate hiking tours (Physical Activity Readiness Questionnaire). The following exclusion criteria were applied: active lifestyle, immunologically mediated chronic conditions or immunodeficiency, severe respiratory diseases, acute or untreated psychiatric disorders, uncontrolled hypertension, uncontrolled metabolic disease, acute infection or fever, diagnosis of or treatment for malignant neoplastic disorders within the last 5 years, arteriosclerotic event <6 months before enrollment, cardiac insufficiency, renal insufficiency, diagnosis or history of alcoholism, current recreational drug use, currently smoking >10 cigarettes/day, orthopedic contraindications for hiking, medication intake >5mg/day prednisone, colchicine, imuran, methotrexate, azathioprine, cyclophosphamide or cyclosporine, intake of weight-loss drugs or preparations and pregnancy. 

For the second sequence of the study, all participants had to be fully vaccinated, recovered or regularly tested for COVID-19. Additionally, rapid COVID-19 antigen tests were performed at arrival. No study participant tested positive throughout the intervention.

#### 2.1.3. Interventions

The participants of both intervention groups spent a seven-day-holiday in Algund (Italy, 46°40′57.5″ N 11°07′19.0″ E), located 350 m above sea level. The region is characterized by its mild, almost Mediterranean climate. All participants were hosted in local hotels and received the same meals. No lifestyle recommendations were given for any group during the non-intervention phase. The activity level of the participants during the intervention was controlled by heart rate monitors (Forerunner 25, Garmin, Olathe, KS, USA).

The hiking group participated in a daily moderate hiking program (Table 1), except for one rest day in the middle of the week. All tours were guided by mountain-hiking-coaches. The “nature group” participated each day in standardized Forest Therapy sessions for 3–4 h (Table 2), assisted by a psychologist. These were characterized by low physical activity. The Forest Therapy was guided by a holistic framework which fosters meaningful connections at three different levels: (1) connection with nature; (2) connection with others; and (3) connection with oneself. Each day was grouped under a certain theme, which is first presented, then discussed in depth and later supported by 3–5 exercises. The session was then ended with a written self-reflection to capture experiences, insights, and thoughts of the day.

#### 2.1.4. Outcomes

Data were collected before the start of the intervention (day 0; T1), after the intervention week (day 7; T2) and again after two (day 60; T3) and six months (day 180; T4) following the intervention. All medical examinations at T1 and T2 were carried out at the Department of Sports Medicine, Tappeiner Hospital Merano (Italy). Follow-up examinations at day 60 took place at the Paracelsus Medical University Salzburg (Austria). The follow-up examination at day 180 was conducted as an online survey. Short-term effects of a single hiking tour and forest therapy session were assessed at day 2 (T1.2, T1.3). Health-related quality of life and quality of relationship were set as primary outcomes. All interventions and assessments are represented in Table 3.

##### Primary Outcomes

Health-related quality of life (HRQOL) was assessed at T1–T4 by the short-form health survey (SF12) and the EuroQol (EQ-5D). The SF12 covers health-related quality of life across the two main dimensions of physical and mental health, as well as a total score [42]. The EQ5D consists of two parts—a descriptive self-assessment in five dimensions, resulting in a health profile index (EQ-5DIndex), and a visual analog scale (EQ-5D-VAS) on which the respondent estimates their current state of health in a range of 0 (worst possible health status) to 100 (best possible health status) [43]. 

In addition to these well-established HRQOL questionnaires, a novel approach to measure HRQOL was used: the intercultural quality of life comic (iQOLC). Developed by the Institute of Ecomedicine at the Paracelsus Medical University Salzburg, the tool assesses HRQOL with a digitally animated comic-based application. It covers 16 items that are rated on a linear scale. The result of each item is displayed in the value range of 1 to 100, whereas higher values represent a better health status. 

The iQOLC is still in the development process. The long-term goal of the iQOLC development is to generate a graphics-based application to validly assess generic health-related quality of life regardless of language, culture and educational background. In addition, disease-specific extensions to the tool are being considered. Within the ANKER Study, the current version of the iQOLC, accessible via www.winterhealth.eu, will be psychometrically validated in the described sedentary population sample, planned as the first step of a comprehensive validation process.

Quality of relationship was evaluated at T1–T4 by the Partnership Questionnaire and the Problem List, which are part of the Partner Diagnostics Questionnaire [44]. The Partnership Questionnaire consists of 30 items, from which three scales (dispute behavior, tenderness and commonality/communication), as well as an overall score, can be formed. Finally, a six-step single item records how unhappy or happy the person currently assesses his/her relationship to be. The Problem List covers 23 problem areas. 

##### Secondary Outcomes—Questionnaires

Nature connectedness was assessed at T1–T4 by the Connectedness to Nature Scale (CNS) and the Nature Relatedness Scale (NRS). The CNS captures the connection with nature with 13 items, which are rated from 1 = “does not apply” to 5 = “applies”. Higher values mean a higher attachment to nature [45]. The NRS assesses closeness to nature over 21 items, which are rated on a scale of 1 = “do not agree” up to 5 = “agree fully”, with higher values indicating a higher closeness to nature [46]. 

Socio-psychological well-being in the sense of flourishing of personality was measured at T1–T4 by the German version of the Flourishing Scale (FS-D). The FS-D consists of eight items, which are answered on a seven-step scale from “I fully agree” to “I absolutely do not agree”. Higher values mean higher socio-psychological well-being [47].

The “Satisfaction with Life Scale” (SWLS) is a one-dimensional questionnaire for recording life satisfaction and was handed out for completion at T1–T4. The SWLS consists out of five items, which are answered on a seven-level Likert scale, with total scores ranging from 5 (lowest satisfaction) to 35 (highest satisfaction). It is standardized for a German population [48]. 

“Subjective impairment” of the participants was assessed at T1–T4 by the complaints list (B-L’). Each item on the B-L’ is rated on a physio scale from 0 = “not at all” to 3 = “strong” [49].

Mindfulness was recorded by the Mindful Attention and Awareness Scale (MAAS). The MAAS comprises 15 items, which are queried on a six-stage scale from 1 = “almost always” to 6 = “almost never”. Higher levels indicate higher levels of mindfulness [50].

The immediate effects of a single hiking/forest therapy session were assessed at day 2 by the “Feeling Scale” (FS), “Felt Arousal Scale” (FAS) and “Mood Scale” (Bf-SR). The FS is a bipolar single-item scale ranging from −5 = “very bad” to 0 = “neutral” to + 5 = “very good” to assess a participant’s pleasure [51]. The FAS is also a single-item scale on which participants rate their level of activation between 1 (low arousal) and 6 (high arousal) [52]. The Bf-SR assesses the current mental state over 24 items, by rating contrary adjectives on a five-point Likert scale [53]. 

The five personality traits of openness, conscientiousness, extraversion, tolerability/agreeableness and neuroticism were assessed at T1–T5 by the BFI-10 questionnaire. Each of the dimensions is rated on a five-point Likert scale from 1 = “not at all” to 5 = “applies fully” [54].

Health locus of control was assessed by the German short version of the questionnaire for the collection of health-related control beliefs (FEGK). The FEGK consists of 10 items, which are answered on a six-point scale with the extreme poles “very correct” to “very wrong” [55]. The latter two (BFI-10, Health locus control) are mainly used for the purpose of assessing discriminant validity of the iQOLC. 

##### Secondary Outcomes—Physiological Parameter

Static balance was assessed at T1–T3 by MFT-S3 Check (Bodywork, Trend Sport Trading GmbH, Großhöflein, Austria). Participants are asked to enter a labile balance disc and are instructed to keep the disc centered. Within two measurement cycles, stability, symmetry and sensorimotor function will be assessed. Balance scores are reported as percent of predicted, based on normative data [56]. 

“Body composition” was measured at T1–T3 by a four-terminal impedance analyzer (BIA-101, RJL Systems; Detroit, Detroit, MI, USA) with two electrodes fixed on the right hand and the other two on the right foot, according to the standard procedure described elsewhere [57]. Data analysis will be performed by Bodygram PLUS software (Akern S.r.l; Pontassieve, Italy). The following parameter will be evaluated: total body water (l), fat mass index (kg/m^2^), fat-free mass index (kg/m^2^), body cell mass index (kg/m^2^), muscle mass index (kg/m^2^) and appendicular muscle mass index (kg/m^2^).

At T1–T3, 12 ml of forearm venous blood was collected in tubes (Vacuette®, Greiner Bio-One GmbH, Austria) according to manufacturer’s guidelines. Serum and plasma samples were stored at minus 80 °C until analysis. Differential blood counts were performed by the Laboratory for Chemical-Clinical Analysis and Microbiology, Tappeiner Hospital Merano (Italy) for T1 and T2, and the University Institute for Medical and Chemical Laboratory Diagnostics of the Paracelsus Medical University Salzburg (Salzburg, Austria) for T3. 

“Fractional exhaled nitric oxide” was measured by NioxMino® (Aerocrine AB, Sweden) according to the ATS/ERS guidelines at T1 and T2 [58]. To assess the immediate effects of the interventions, additional fractional exhaled nitric oxide measurements were performed before and after a single hiking/forest therapy session on day 2. 

“Anthropometric measures” (height, weight, waist and hip circumference) were performed according to the WHO guidelines [59]. “Height” was measured by a wall-mounted stadiometer. BMI and waist–hip ratio were calculated.

The “Chester step test” was used to assess aerobic fitness at T1–T3. During Chester step test, participants are asked to step on and off a low step at a defined rate, which is set by a metronome. Every two minutes, the heart rate and exertion level are recorded. The test continues until the participant reaches 80% of its maximum predicted heart rate [60]. Before and after the step test, the peak expiratory flow is measured in triplicates by a peak flow meter (Mini-Wright peak flow meter), and the best value is recorded. 

Transepithelial water loss was measured by a Tewameter® TM 300 (Courage + Khazaka electronic GmbH, Köln, Germany) at T1–T3. Skin hydration was measured by a Corneometer® CM 825 (Courage + Khazaka electronic GmbH, Köln, Germany) at T1–T3. 

##### Environmental Monitoring

In addition to personal data, environmental parameters, such as particulate matter, volatile organic compounds or microbiome profiles, are also collected as part of the study. Environmental parameters were measured at the forest therapy site, at a selected point of the hiking tours, and at a control site at the city of Meran. Air quality, including particulate matter 1 µg/m^3^, 2.5 µg/m^3^, 10 µg/m^3^ and volatile organic compounds, was measured by a portable air quality monitor (Atmotube PRO, Atmotech Inc., San Francisco, CA, USA). The radon concentration was measured with a Radon/Thoron Monitor 1688 (SARAD GmbH, Dresden, Germany). Nanoparticles were measured with a multimeric nanoparticle detector (Partector 2, naneos particle solutions gmbh, Windisch, Switzerland). The density of air ions was measured by an air ion counter (AIC2, AlphaLab Inc., Salt Lake City, UT, USA). Furthermore, microbiome samples were collected with sterile swabs and an air sampler (Coriolis Compact, Bertin Technologies SAS, Montigny-le-Bretonneux, France). 

**Figure 1:**
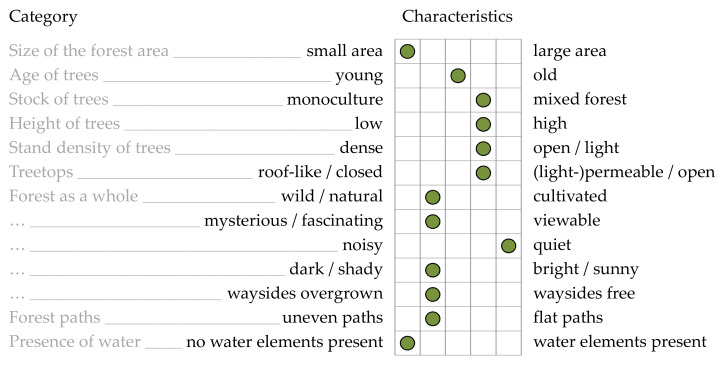
Exemplary forest profile based on presented evaluation approach (Source: own illustration).

Finally, an evaluation of the forest described in the Introduction was carried out. An example of such a forest profile is given below (Figure 1). The survey form for assessing a forest can be found in full length in the Appendix A (see Table A1). The presented study protocol will focus mostly on human–nature interaction.

#### 2.1.5. Sample Size

Sample size was estimated using health-related quality of life data from a former intervention study [61] (ISRCTN18092043). The sample size for the study was approximated with the statistical packages G*Power (G*Power Ver. 3.0.10, Franz Faul, Universität Kiel, Germany). The sample size was estimated for an ANOVA with fixed effects, special effects, main effects and interactions with the following input parameters: effect size f = 0.38, type I error α = 0.05, power 1-β = 0.85, number of groups = 2, degree of freedom = 2. The required sample size for getting a power of at least 85% was estimated to be 39 persons per group.

#### 2.1.6. Recruitment

Participants were recruited via a webpage (https://www.klimatherapie.eu/, accessed on 22 January 2021) and advertisements posted on social media channels. An eligibility check was designed as a two-step process: in a preliminary online form, sociodemographic data (age, relationship status), BMI and activity level were (International Physical Activity Questionnaire—Short Form, two questions) assessed. Eligible persons were invited to fill out a second online form, which evaluated general health status (PHQ-9), nature connectedness (NRS-6) and physical activity readiness (PAR-Q).

### 2.2. Assignment of Interventions

Randomization was performed by an open-source add-in (Daniel’s XL Toolbox, Ver. 7.2.7) for the Microsoft Excel® spreadsheet software in blocks of two (pairwise allocation of couples), with age, general health status (PHQ-9), nature relatedness (NRS-6), BMI, activity level (IPAQ-SF) and relationship duration as allocation criteria [62]. As allocation method, the Kullback–Leibler divergence method was used. During randomization, patient data were anonymized by sequential numbers. Assignment to the interventions was communicated at day 0 after baseline examinations. Recruitment, eligibility check and assignment of participants to the interventions were performed by the same research scientist. Sequence generation, randomization and all following statistical analyses were performed by independent research scientists. Due to the intervention type, no blinding was planned.

### 2.3. Data Collection, Management, and Analysis

#### 2.3.1. Data Collection Methods

Trained research scientists collected all data. Once a subject was enrolled for the ANKER Study, the researchers made every effort to avoid possible sample loss. Questionnaires at T0–T2 were paper–pencil-based and were digitalized by trained research scientists. Questionnaires at T3 were based on an online survey tool. To avoid missing data points, all answers were set as mandatory. No further data were collected from participants who were excluded from the study.

#### 2.3.2. Data Management

Data were anonymized by four-digit ID numbers. The master list, which contains the assignment of the IDs to the personal data, is stored on a data server of the Paracelsus Medical University Salzburg (Austria) and is only accessible to the research scientist in charge of recruitment, eligibility check and assignment. Data from medical examinations and surveys are stored in spreadsheet files. Only authorized researchers have access to the data. Participants can access their personal data after completing the study.

#### 2.3.3. Statistical Methods

Analyses were performed in accordance with the intention-to-treat principles, and the reporting adheres to the CONSORT statement, including CONSORT flow chart [63]. All statistical analyses were executed using the R-GNU software environment (General Public License, R Foundation for Statistical Computing, Vienna, Austria). Statistical significance was set at the level of a <0.05 for all tests. Randomly missing values were replaced using the standard procedure, last outcome carried forward. The Shapiro–Wilk test was applied to check for normal distribution. Depending on data distribution, parametric or non-parametric tests were be applied. Participant data were compared in terms of baseline data, including outcome variables, as well as demographics (unpaired Student’s T-test or Wilcoxon test). Changes over time and between the interventions were either analyzed by linear mixed models or F1-LD-F1 models from the nparLD package [64]. In both cases group, time and group*time interaction effects were assessed. Furthermore, a post hoc sample size calculation based on bootstrap simulation was performed [65].

Psychometric analysis of the iQOLC was performed to test reliability, validity and responsiveness to change. To examine the fit of the assumed three-factor structure of the iQOLC (physical, psychological and social health), a confirmatory factor analysis was conducted, and the following model fit indices were calculated: chi-square, root mean square error of approximation, standardized root mean square residual and comparative fit index.

### 2.4. Monitoring

During all phases of the ANKER Study, the study coordinator supervised the study. In regular performance audits, the entire research team ensured that all participants met the inclusion criteria and that all performed the activities proposed by the study protocol. No data monitoring committee was installed. No interim analyses were performed. Although clinical evaluations and the planned hiking/forest therapy program are considered as having a low personal risk, adverse events would have been monitored and recorded by the researchers and reported to the research coordinator of the study and to the Ethics Committee of Bolzano, Italy.

### 2.5. Ethics and Dissemination

The study protocol was approved by the Ethics Committee of Bolzano (Comprensorio Sanitario di Bolzano, reference number: 18–2019, date of approval 2019/03/13). In the case of study protocol modifications, the approval of the Ethics Committee would have been sought immediately. Any changes were communicated to the participants and trial registry. Informed consent was obtained from all participants prior to enrollment by the researcher in charge of recruitment and eligibility check. All employees pledged confidentiality with their signature. The master list containing the assignment of the IDs to the personal data was accessible to authorized persons only. All statistical analyses were performed with anonymized data. In all publications and presentations, the personal data of individuals were not traceable. Trial data and documents from the ANKER Study were stored in the archives of the Paracelsus Medical University Salzburg, Institute of Ecomedicine, Austria, and will be made available on request after publication. The results of this protocol study will be published in peer-reviewed journals, as well as at national and international conferences. The authors declare that they have no competing interests.

## 3. Discussion

The 21st century is characterized by a rapid growth of urban agglomerations, signified by externally structured and everyday life organizations in gray, geometric environments. This has led to the rediscovery of the forest as a “healing resource” for urban society. Forests promise peace, orientation, freedom, deceleration and fulfill our longing for authentic nature experience. Forests reduce stress outside of everyday life [66].

Given improvements to the validity of studies on Exposome and to the effects of individual external factors on human health, especially to natural factors, such as forests, such effects can be much better integrated into public health policies. To this purpose, this paper provides a variety of approaches, which address important research gaps. Certainly, these need to be examined in much more detail in the future.

The most important aspect relates to the selection of health outcome parameters of nature-based clinical intervention trials. Most studies in forest therapy put their research focus on surrogate parameters, such as natural killer cells count and activity or (short-term) reduced blood pressure and stress hormones [67]. An improvement of these parameters may only suggest a potential preventive health effect, but it does not show a clinically significant patient benefit. On the other hand, the selected primary outcomes of the ANKER Study (health-related quality of life, relationship quality) represent patient-centered clinical endpoints, thus indicating clinically meaningful changes and a direct patient benefit [68]. As a corollary, the ANKER Study also takes into account the emerging necessity of considering humans as integrated, feeling and active beings and not simply as biological organisms [69]. Moreover, it points to the demand for a stronger integration of patient-reported outcomes in clinical trials [70].

Another aspect concerns the role of the control group. The intention of the control group should not be to achieve the greatest possible difference in health effects. To extract the specific influence of a forest on human health, the control group must be carried out in the same spatial setting, namely the forest. This is the only way to come closer to the actual impact of forests on human health. In this respect, the authors propose to use the survey questionnaire used here to determine the forest setting in future studies. Furthermore, this will leverage the comparability of studies. Ultimately, it will help in determining the actual influence of forests and the conducted activities on health.

Only when a more precise picture of the health effects of forests together with corresponding activities emerges can forests be seriously and reasonably used in a medical sense. Additionally, only then does it make sense, from the authors’ point of view, to assign a specific health function to individual forest areas, as already practiced in, for example, so-called “healing forests” [71].

In this context, the findings on the health effects of forests can be used, for example, to increase the general importance of nature-based health prevention. On the one hand, evidence-based health benefits of forests could become more important for individual health prevention and thus also help reduce the pressure on the public health care system [72]. On the other hand, such an approach can also be seen as a direct extension of a public health care system, which integrates prevention as an important factor of human health [73]. In this context forests play a special role as one of many natural healing resources, since forest areas, in contrast to waterfalls, for example, are more frequently available and thus more easily accessible by a large part of the population. For example, almost one-third of the world’s land mass is covered by forests, which corresponds to about 0.5 hectares of forest per capita [74]. Of course, there is a relatively strong inequality of distribution. However, if we consider only forests within the European Union, where the best conditions for such an approach probably exist in the form of public health systems, and where the proportion of forest areas even exceeds 40%, this inequality almost completely disappears [75].

Another benefit can be seen in the opportunities to develop nature-based health tourism offers that provide a proven, evidence-based, added health value. Thereby, the fundamental demand for nature experiences can be loaded with the added value of a higher medical evidence. This, again, perfectly fits with the current trend of an increased health consciousness in society [76]. When combined with other trends in tourism, such as the search for more regionality and more authenticity, and thus for more resonance experiences [77], innovative and market-oriented products can be created.

## 4. Conclusions

This paper shows that further analyses of the health impact of forests is much needed, particularly regarding a more rigorous analysis of different natural areas and natural resources and of their possible integration into existing public health care systems. The example of Algund shows that health effects of nature are becoming more and more relevant for public authorities. Additionally, one can assume that the initial implementation of such an approach at community level would be less difficult than at national or EU level. Nevertheless, such projects should be realized on different levels in the future and, at best, should also be funded by the public sector. Ultimately, this study reveals that transdisciplinary research projects do not only offer high added value for scientific research and the regions involved as partners, but that essential transfers are achieved for society at large.

## Figures and Tables

**Table 1:** Exercise program of the hiking group.

	Distance (km)	Altitude (m)	Duration (h)
Day 1	12.6	334	04:07
Day 2	7.5	298	03:03
Day 3	10.0	639	04:01
Day 5	9.8	569	03:38
Day 6	5.9	680	02:37

**Table 2:** Thematic schedule of the nature group.

	Theme	Content	Activities
Day 1	Mindfulness and relaxation	Importance of mindfulness and relaxation in personal and work life, explanation of how nature can be used to foster and induce mindfulness and psycho-physiological relaxation [38];	Nature-based mindfulness practices, e.g., walking meditation
Day 2	Connection to nature	Importance of health benefits of nature connection; “forest bathing” as a formal method to strengthen the bond between oneself and one’s natural environment [39];	“Forest bathing” activities, e.g., mindfully breathing with a tree
Day 3	Social connections	Importance of social relationships, methods to calm down the nervous system to improve the social engagement system, which allows for connecting with one another better [40];	Interpersonal mindfulness exercises, e.g., natural artwork
Day 5	Connection to self	Importance of self-awareness, different aspects of oneself as a critical factor for mental well-being [41], how to use nature as a tool to initiate self-reflective processes;	e.g., medicine walk, invitation to communicate with nature
Day 6	Goal setting and next steps	Goal setting, behavioral change, self-regulation, transformation of the practices and exercises learned in this program into lasting habits	Nature-based mindfulness practices

**Table 3:** Participant timeline showing time schedule of enrollment, interventions and assessments of participants. Abbreviations: BMI—Body Mass Index, IPAQ-SF—International Physical Activity Questionnaire Short Form, FEGK—Questionnaire for the Collection of Health-Related Control Beliefs, VAS—Visual Analog Scale.

	STUDY PERIOD							
	Enrollment		Allocation	Post-Allocation				
TIMEPOINT	T_-2_	T_-1_	T0 Baseline	T_1_ Day 0	T_1.2_ Day 2	T_2_ Day 7	T_3_ Day 60	T_4_ Day 180
ENROLLMENT								
Eligibility screen—step 1 Sociodemographic data | BMI IPAQ-SF | 2 Questions	x							
Eligibility screen—step 2 Medical history Physical Activity Readiness Questionnaire Patient Health Questionnaire (PAR-Q) Nature Relatedness Scale 6 (NRS-6)		x						
Informed consent		x						
Group allocation			x					
INTERVENTIONS								
Mountain hiking				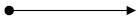				
Forest Therapy				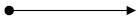				
ASSESSMENTS								
Primary Outcomes Short Form Health Survey (SF-12) | iQOLC Euro Quality of Life Questionnaire (EQ-5D) Partnership Questionnaire | Problem List				x		x	x	x
Secondary Outcomes Connectedness to Nature Scale | NRS-6 Mindful Attention and Awareness Scale Flourishing Scale | Complaints List Satisfaction with Life Scale | IPAQ-SF 10 Item Big Five Inventory | FEGK				x		x	x	x
Secondary Outcomes BMI | Skin quality Chester step test | Peak Flow Balance—MFT-S3 Check Differential blood count				x		x	x	
Waist–hip ratio |Exhaled nitric oxide				x		x		
Short-term effects Feeling Scale | Felt Arousal Scale | Mood Scale | VAS |Exhaled nitric oxide					x			
Control parameter HR monitoring				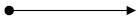				

## Data Availability

The data presented in this study are available on request from the corresponding author.

## Abbreviations

BFI-10	10 Item Big Five Inventory
Bf-SR	Mood Scale
BIA	Bio Impedance Analysis
B-L’	Complaints List
BMI	Body Mass Index
CNS	Connectedness to Nature Scale
EQ-5D	Euro Quality of Life Questionnaire
FAS	Felt Arousal Scale
FEGK	Questionnaire for the Collection of Health-Related Control Beliefs
FS	Feeling Scale
FS-D	German Version of the Flourishing Scale
HRQOL	Health-Related Quality of Life
IPAQ-SF	International Physical Activity Questionnaire Short Form
iQOLC	Intercultural Quality of Life Comic
MAAS	Mindful Attention and Awareness Scale
nparLD	Nonparametric Longitudinal Data Analysis
NRS-6	Nature Relatedness Scale 6
PAR-Q	Physical Activity Readiness Questionnaire
PFB	Partnership Questionnaire
PFD	Partner Diagnostics Questionnaire
PHQ-9	Patient Health Questionnaire
PL	Problem List
SF-12	Short Form Health Survey
SWLS	Satisfaction with Life Scale
VAS	Visual Analog Scale

## Appendix A

**Table A1:** Survey form for the forest profile.

**Scheme 1.**				Filled by:		
Date:				Weather:		
Location/forest:				Route:		
Duration:		Distance in km:			Altitude in m:	
Scale explanation						

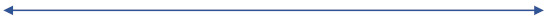

Guidance* Characteristic X	1 = is fully true	2 = rather true	3 = partially	4 = rather true	5 = is fully true	Guidance* Characteristic Y
**Characteristics forest/trees**						
<200 hectares	Size of the forest area					>1000 hectares
small area	1	2	3	4	5	large area
<10 years	Age of trees					>50 years
young	1	2	3	4	5	old
	Stock of trees					
monoculture	1	2	3	4	5	Mixed forest
<5 meters	High and structure of the trees					>20 meters
low	1	2	3	4	5	high
single stage	1	2	3	4	5	multistage
Stand density of the trees						
dense	1	2	3	4	5	open/light
Structure of treetops						
low-hanging	1	2	3	4	5	high
not sprawling	1	2	3	4	5	sprawling
roof-like/closed	1	2	3	4	5	(light-)perme-able/open
Forest as a whole						
wild/natural	1	2	3	4	5	cultivated
mysterious/fascinating	1	2	3	4	5	viewable
relaxing	1	2	3	4	5	stimulating
cool	1	2	3	4	5	warm
dirty (garbage)	1	2	3	4	5	clean (garbage)
loud	1	2	3	4	5	quiet
lonely	1	2	3	4	5	full/socially connecting
unsafe	1	2	3	4	5	safe
dark/shady	1	2	3	4	5	bright/sunny
Other vegetation						
monotonous	1	2	3	4	5	biodiverse
colorless	1	2	3	4	5	colorful
**Survey form forest (2/2)**						
Scale explanation						

Characteristic X	1 = is fully true	2 = rather true	3 = partially	4 = rather true	5 = is fully true	Characteristic Y
**Characteristics forest paths/forest floor**						
Condition/structure forest paths						
waysides overgrown	1	2	3	4	5	waysides free
slim	1	2	3	4	5	wide
twisty	1	2	3	4	5	straight
plain	1	2	3	4	5	steep
uneven	1	2	3	4	5	flat
hard	1	2	3	4	5	soft
designed	1	2	3	4	5	natural
Condition/structure forest floor						
bare	1	2	3	4	5	overgrown
impassable	1	2	3	4	5	accessible
**Other characteristics of the forest**						
no water elements *1 present	1	2	3	4	5	Water elements present
no views/sceneries present	1	2	3	4	5	views/sceneries present
no natural resting places *2 present	1	2	3	4	5	natural resting places present
not barrier-free	1	2	3	4	5	barrier-free

* Guidance is provided in a few cases for orientation purposes. Essentially, however, it is a rather subjective evaluation in the sense of “What applies predominantly to the forest?”. *1 Water elements: creek, river, lake, waterfall, etc. *2 Natural resting places: Moss, tree stumps, meadows, hills/clearings, etc.
